# Focused ultrasound as a treatment modality for gliomas

**DOI:** 10.3389/fneur.2024.1387986

**Published:** 2024-05-15

**Authors:** Divine C. Nwafor, Derrick Obiri-Yeboah, Faraz Fazad, William Blanks, Melike Mut

**Affiliations:** ^1^Department of Neurosurgery, University of Virginia, Charlottesville, VA, United States; ^2^Department of Neurological Surgery, Cleveland Clinic Lerner College of Medicine of Case Western Reserve University, Cleveland, OH, United States; ^3^Department of Neurosurgery, Rockefeller Neuroscience Institute, West Virginia University, Morgantown, WV, United States

**Keywords:** focused ultrasound, glioma, glioblastoma, clinical trials, HIFUS, LIFUS, blood–brain barrier

## Abstract

Ultrasound waves were initially used as a diagnostic tool that provided critical insights into several pathological conditions (e.g., gallstones, ascites, pneumothorax, etc.) at the bedside. Over the past decade, advancements in technology have led to the use of ultrasound waves in treating many neurological conditions, such as essential tremor and Parkinson’s disease, with high specificity. The convergence of ultrasound waves at a specific region of interest/target while avoiding surrounding tissue has led to the coined term “focused ultrasound (FUS).” In tumor research, ultrasound technology was initially used as an intraoperative guidance tool for tumor resection. However, in recent years, there has been growing interest in utilizing FUS as a therapeutic tool in the management of brain tumors such as gliomas. This mini-review highlights the current knowledge surrounding using FUS as a treatment modality for gliomas. Furthermore, we discuss the utility of FUS in enhanced drug delivery to the central nervous system (CNS) and highlight promising clinical trials that utilize FUS as a treatment modality for gliomas.

## 1 Introduction

Central nervous system (CNS) tumors are diverse tumors with distinct and variable intrinsic characteristics. Of this broad category, roughly 28.8% are comprised of tumors with neuroepithelial origin, with an incidence rate of 5.56 per 100,000 persons. Glioblastoma, the most common and one of the most aggressive primary glial tumors has an incidence rate of 2.52 per 100,000 persons (1). In other words, there are over 10,000 new glioblastoma diagnoses in the United States annually. Notably, these tumors are not homogenously distributed among the population; they have higher incidences in specific ethnicity subgroups (2).

Over the past decades, much research has been conducted on the epidemiology of primary CNS tumors, including glial tumors; however, advancement has yet to be made in novel treatment modalities that significantly extend the duration and quality of life in patients suffering from these tumors. Epidemiology and early detection strategies are essential to studying any malignant process; however, due to the aggressive nature of certain subsets of these tumors, including glioblastoma, their clinical impact is limited (3). The current mainstay treatment of glioblastoma includes resection, radiation therapy, and chemotherapy (4, 5). Even with gross total resection and maximal radiation therapy and chemotherapy, survival rates are less than 2 years for most patients. New surgical tools, such as 5-aminolevulinic acid (5-ALA) and volumetric MRI evaluation, can help maximize the extent of surgical resection; however, they still offer relatively low survival benefits (6).

Ultrasound technology has been studied for its ablative effects on the brain since the 1950s. Still, significant limitations, such as the need for a craniotomy window and difficulty with targeting precision, prevented it from gaining mainstream attention until decades later. Elias et al. conducted the first pilot study of focused ultrasound thalamotomy for essential tremors, showing that this was a safe and effective treatment (7). Thereafter, the indications for FUS have continued to grow, including treating Parkinsonian tremors/rigidity or managing many neuropsychiatric conditions (8). New indications for FUS beyond functional neurodegenerative disease conditions remain under study; however, data from several neurosurgical laboratories have begun to provide new evidence that supports the use of FUS in treating CNS glioma.

Additionally, the effect of novel drugs, such as immune checkpoint inhibitors, is significantly dampened secondary to the difficulty of such inhibitors permeating the intact blood–brain barrier. To address this difficulty, blood–brain barrier opening utilizing FUS provides a novel solution that allows novel chemo/immunotherapy to reach their desired targets and more effectively treat CNS tumors. This mini-review article discusses focused ultrasound and its novel applications in treating gliomas. Finally, we highlight promising clinical trials utilizing FUS as a glioma treatment modality.

## 2 Types and mechanisms of FUS

“Ultrasound” refers to a wave possessing a frequency beyond human hearing, typically *f* > 20 kHz (9). Ultrasound imaging has been employed for decades to visualize anatomic structures in the medical field—for example, prenatal or transthoracic echocardiogram. However, recent research focuses on the applications of ultrasound for therapeutic purposes. Ultrasound imaging converts electrical energy into mechanical energy by transmitting acoustic waves through a transducer (10). These waves penetrate tissues –such as skin and muscle–to reach molecules within deeper structures. Thereafter, the waves can either be absorbed, scattered, or reflected. When a molecule with a suitable frequency encounters the ultrasound waveform, energy transfer occurs; this concept is known as resonance (11).

Interventional ultrasound for ablative and non-ablative purposes utilizes the same principles governing ultrasound imaging. The waves produced can interfere constructively or destructively. These unique properties underlie the use of ultrasound waves as a diagnostic and therapeutic tool by controlling the interference of ultrasound waves (12). Constructive interference occurs when two or more waves meet, and their peaks or troughs overlap. The resultant wave in constructive interference results in a final wave with a greater amplitude than each individual wave. Conversely, destructive interference occurs when the consequent wave from two or more waves results in an overall final waveform with a smaller amplitude due to waves canceling out (13). In general, FUS aims to allow in-phase waves to converge at the therapeutic target location (12, 14).

FUS is an ultrasound modality that utilizes a concave transducer to converge ultrasound waves into a focused beam. FUS can be categorized as higher-frequency (HIFUS) and low-frequency (LIFUS). HIFUS is often used to ablate specific targets, whereas LIFUS is often used to improve drug delivery to specific targets (14). HIFUS beam intensities are typically between 100–10,000 with the objective of the thermal ablation of tissue, while LIFUS ranges from 0.125–3 W/cm^2^ (14). The applications of HIFUS for functional neurosurgery have been vast, ranging from ablating the globus pallidus internus in Parkinson’s disease to ablating an epileptic hippocampal focus (15, 16). Delivery of FUS waves is affected by skull fat and bone. FUS wave delivery can be enhanced by applying gasless water between the ultrasound transducer and the scalp. Concurrently, this also minimizes thermal damage. Magnetic resonance-guided imaging (MRgFUS) is critical in identifying/planning the target area and monitoring ablation size. If the target area is heated beyond 56°C for a few seconds, thermo-ablation occurs via protein denaturation and coagulative necrosis. MRgFUS allows monitored and controlled real-time thermometry, which enables immediate evaluation of treatment response (17).

**Figure 1:**
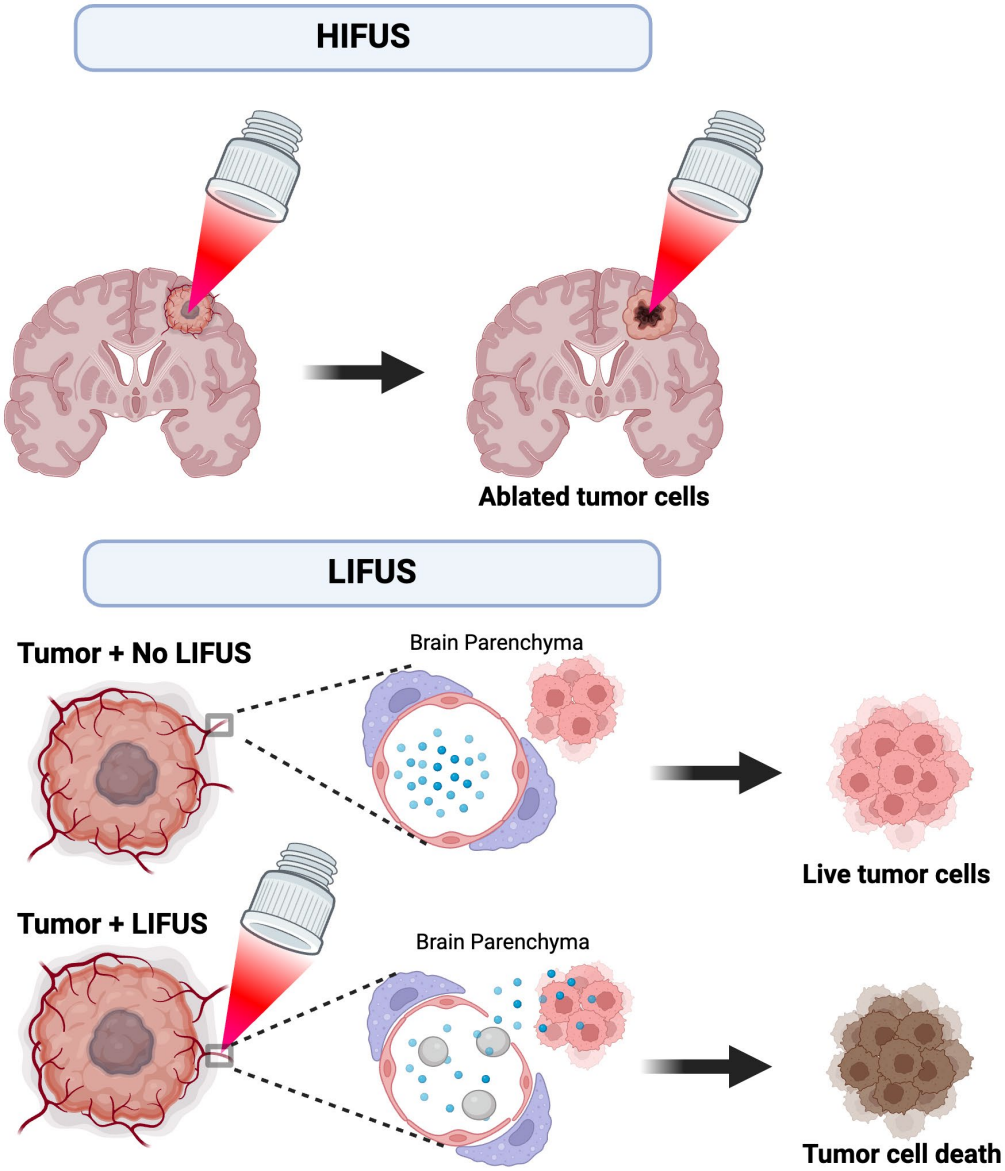
Proposed mechanism and application of HIFUS and LIFUS. HIFUS utilizes non-invasive, high-energy focused ultrasound waves to thermally ablate tumors. Alternatively, LIFUS utilizes lower ultrasound wave energy and microbubbles (grey) to disrupt the blood–brain barrier (BBB) and transiently allow chemotherapy/immunotherapy (blue) to permeate into the brain parenchyma. Ultimately, these treatments reach the tumor cell targets and promote cell death.

Wave energy is created by passing an electrical current through a transducer to achieve thermoablation with HIFUS. Continuous high-pressure waves are then directed at a small target point, resulting in tissue destruction via a thermal effect. LIFUS ultrasound utilizes injected exogenous microbubbles to open the blood–brain barrier (BBB) by applying ultrasound non-thermal waves that promote microbubble size change/expansion – this is referred to as “stable cavitation.” The perturbations in the size of the microbubbles promote BBB opening (18). Recent studies suggest BBB closure occurs within 48 h after LIFUS without causing injury or harm to the patient (19). Figure 1 demonstrates the proposed mechanisms and application of HIFUS and LIFUS in treating CNS tumors.

## 3 FUS in the management of glioma

The BBB is a selective dynamic barrier composed of endothelial cells with tight junction proteins, astrocytic end-feet processes, surrounding pericytes, and basal lamina that plays a critical role as a semi-permeable interface between the systemic circulation and brain parenchyma. In health, the BBB is important as it protects the brain from harmful toxins/molecules and inflammatory immune cells - and ultimately maintains cerebral homeostasis by regulating nutrients via modulation of proteins and enzymes found in several cell types of the BBB (20). However, in many disease states, such as CNS tumors, the selectivity of the BBB presents a challenge for therapeutic drugs to reach desired targets. Traditionally, neurosurgeons have mitigated this problem by increasing the dose of the drug – which worsens the adverse effect. Another option is intraventricular or intrathecal drug delivery. Though the effects of these two drug delivery methods strategies are pronounced compared to intra-arterial drug delivery, they do not effectively address the lesion of interest. Furthermore, specificity is significantly reduced (21).

In the early stages of glioma growth, tumor cells initially resemble the BBB, and as they continue to proliferate and progress, they form a new barrier known as the blood–brain tumor barrier (BBTB). The BBTB is distinct from the BBB in that it is characterized by an aberrant distribution of healthy BBB cell types (e.g., pericytes), astrocytic end-feet loss, neuronal dysfunction, increased expression of proteins that encourage drug transport efflux, and a heterogenous permeability between the tumor core and periphery (22–24). The BBB and BBTB limit the entry of chemotherapies and immunotherapies to reach CNS tumor targets (25). Traditionally, hyperosmotic agents such as mannitol have increased BBTB permeability to therapeutic agents when managing glioma and other CNS tumors (26, 27). Though mannitol has provided some success, it has many drawbacks that include limited BBTB opening (15 min), non-selective BBTB opening that can also affect healthy BBB, limited permeability to larger molecules, and systemic effects such as electrolyte abnormalities, injury to kidneys, and worsening of heart failure (28). Numerous strategies, including the use of prodrug formulations, chemical barrier disruption, intraarterial injection, surgical circumvention, thermotherapy, etc., have all been investigated as ways to circumvent the BBB and BBTB but have had limited success (25).

FUS is a safe thermotherapy modality shown to ablate CNS tumors directly or enhance drug delivery across BBB/BBTB for tumor treatment. Other forms of thermotherapies include radiofrequency microwaves, laser-interstitial thermotherapy (LITT), and magnetic disruption (25, 29). These modalities work via induction of intracranial hyperthermia, which causes potentiation of radiotherapy and chemotherapy. Additionally, the resulting hyperthermia demonstrates preferential glioma cell cytotoxicity and increases BBB permeability and tumor cell death through heat-shock protein-mediated cytotoxicity (25, 29, 30). FUS holds the most promise of the various thermotherapy modalities due to its noninvasive nature, efficacy, and ease of performing. Experiments in rat models have demonstrated a higher (38.6%) CSF-to-plasma ratio for temozolomide transferred with focused ultrasound relative to 22% observed in control modalities (25, 31). Like other modalities in this treatment class, FUS thermos-mechanically disrupts the BBB; however, a combination of LIFUS with intravenous injection of albumin-coated octafluropropane microbubbles has been shown to produce only a transient opening of the BBB, thereby significantly decreasing permanent tissue damage (25, 32, 33).

FUS–mediated BBB disruption has been used successfully to deliver numerous agents in animal models including doxorubicin (34, 35), trastuzumab (36), temozolomide (31), interleukin-12 (37), anti-programmed cell death-ligand-1 antibody (38), poly (ethylene glycol) - poly (lactic acid) nanoparticles (39), adeno-associated virus (40), and AP-1 lipoplatin (41). Recent studies have quantified 2000kD as the upper particle size limit for successful FUS-mediated BBB transfer – a size limit encompassing numerous therapeutic agents (25, 42, 43). Beyond the mechanical opening of the BBB/BBTB to allow for enhanced drug delivery, FUS has also been shown to decrease the expression of efflux transporters, reduce junctional proteins, and modify the dispersion of nanoparticles in the extracellular space (44–47). There is growing translational research evidence for successful BBB disruption using FUS, as discussed above, which has led to numerous clinical trials further to assess the role of FUS in glioma treatment, as detailed in Table 1.

**Table 1:** Summary of findings for completed and ongoing clinical trials utilizing fus to treat glioma/glioblastoma.

Trial name	Phase	Status	Summary	Treatment	Trial identifier	Publications
Non-Invasive Focused Ultrasound (FUS) with Oral Panobinostat in Children with Progressive Diffuse Midline Glioma (DMG)	1	Ongoing	Children with progressive diffuse midline gliomas (DMG) treated with oral Panobinostat using FUS with microbubbles and neuro-navigator-controlled sonication.	FUS + Chemotherapy	NCT04804709	N/A
FUS Etoposide for DMG - A Feasibility Study	1	Ongoing	Children with progressive DMG treated with oral etoposide using focused ultrasound with microbubbles.	FUS + Chemotherapy	NCT05762419	N/A
A Phase 2 Study of Sonodynamic Therapy (SDT) Using SONALA-001 and ExAblate 4,000 Type 2.0 in Patients with Diffuse Intrinsic Pontine Glioma (DIPG)	2	Ongoing	Sonodynamic therapy (SDT) using SONALA-001 and ExAblate Type 2.0 device and to determine the maximum tolerated dose (MTD) or recommended phase 2 dose (RP2D) of MR-Guided Focused Ultrasound (MRgFUS) energy in combination with SONALA-001.	FUS	NCT05123534	N/A
Study of SDT Therapy in Participants with Recurrent High-Grade Glioma (HGG)	0	Ongoing	Ascending energy doses of SDT utilizing the MRgFUS combined with IV aminolevulinic acid (ALA) to assess safety and efficacy in participants with recurrent HGG. Participants who are scheduled for resection will be administered IV ALA approximately six to seven (6–7) hours prior to receiving SDT.	FUS	NCT04559685	N/A
Assessment of Safety and Feasibility of ExAblate BBB Disruption in Gliobastoma(GBM) Patients	N/A	Completed	Evaluate the safety of the ExAblate Model 4,000 Type 2.0 used as a tool to disrupt the BBB in patients with high grade glioma undergoing standard of care therapy. Findings demonstrated that MRgFUS can safely open BBB in GBM patients.	FUS	NCT04998864	N/A
Assessment of Safety and Feasibility of ExAblate Blood–Brain Barrier (BBB) Disruption	N/A	Ongoing	Evaluate the safety of the ExAblate Model 4,000 Type 2 used as a tool to disrupt the BBB in patients with high grade glioma undergoing standard of care therapy.	FUS	NCT03551249	N/A
ExAblate Treatment of Brain Tumors	N/A	Completed; results not published	A Study to Evaluate the Safety and Feasibility of Transcranial MRI-Guided Focused Ultrasound Surgery in the Treatment of Brain Tumors	FUS	NCT01473485	N/A
Assessment of Safety and Feasibility of ExAblate BBB Disruption for Treatment of Glioma	N/A	Completed	First proof of concept study demonstrating that MRgFUS enriches systemic circulating brain-derived biomarkers via a process known as liquid biopsy.	FUS	NCT03616860	PMID: 33693781
BBB Disruption Using ExAblate Focused Ultrasound with Doxorubicin for Treatment of Pediatric diffuse intrinsic pontine glioma (DIPG)	Phase 1	Ongoing	Evaluate the safety and efficacy of targeted BBB disruption with ExAblate Model 4,000 Type2.0/2.1 in combination with Doxorubicin therapy for the treatment of DIPG in pediatric patients	FUS + Chemotherapy	NCT05630209	N/A
BBB Disruption for Liquid Biopsy in Subjects with GBM	N/A	Ongoing	Evaluate the safety and efficacy of targeted BBB disruption with ExAblate Model 4,000 Type 2.0/2.1 for liquid biopsy in subjects with suspected GBM	FUS	NCT05383872	N/A
Efficacy and Safety of NaviFUS System add-on Bevacizumab in Recurrent GBM Patients	N/A	Completed	To investigate the efficacy and safety of FUS add-on bevacizumab in recurrent GBM patients. Findings demonstrated that MRgFUS can safely open BBB and enhances bevacizumab delivery which significantly decreased tumor growth and increased median survival.	FUS + Chemotherapy	NCT04446416	PMID: 27192459
SDT in Patients with Recurrent GBM	Phase 1	Ongoing	Evaluate the safety and feasibility of combining an investigational drug called 5-ALA with neuronavigation-guided low-intensity focused ultrasound (LIFUS) for patients who have recurrent GBM. SDT will take place prior to surgery for recurrent GBM.	FUS	NCT06039709	N/A
Safety of BBB Disruption Using NaviFUS System in Recurrent GBM Patients	N/A	Completed; results not published	Evaluate the safety and find the tolerated ultrasound dose of transient opening of the BBB by using the NaviFUS System in recurrent GBM patients.	FUS	NCT03626896	N/A

ALA, Aminolevulinic acid; BBB, Blood–Brain Barrier; DIPG, Pediatric diffuse intrinsic pontine glioma; DMG, Diffuse Midline Glioma; FUS, Focused Ultrasound; GBM, Gliobastoma; HGG, High-Grade Glioma; LIFUS, low-intensity focused ultrasound; MRgFUS, MR-Guided Focused Ultrasound; SDT, Sonodynamic Therapy; TMZ, Temozolomide.

In conjunction with BBB/BBTB opening, FUS use in managing CNS tumors has allowed for better sampling of tumor-specific biomarkers secreted into systemic circulation during FUS-associated BBB/BBTB opening. This process is referred to as liquid biopsy (48). Analyses of these biomarkers may enable early detection, predict recurrence, and assess treatment response. Alternatively, FUS can be used to achieve CNS tumor ablation via hyperthermia. McDannold et al. and Coluccia et al. and others have demonstrated the successful utility of HIFUS ablation in managing CNS tumors (49, 50). Yet, the definitive role of HIFUS ablation in the management of glioma remains to be clinically validated, though preclinical studies have demonstrated success in glioma treatment (14). Interestingly, preclinical data also suggest that the hyperthermia from HIFUS ablation may sensitize glioma cells to radiation therapy (51).

## 4 Adjuncts to FUS in the treatment of glioma

### 4.1 Sonodynamic therapy (SDT)

Sonodynamic therapy (SDT), a treatment modality similar in mechanism to photodynamic therapy (PDT), is a promising alternative treatment being investigated for glioma treatment. In PDT, a light-activated photosensitizer generates reactive oxygen species (ROS), facilitating cytotoxic effects on neoplastic cells. While effective, PDT is limited to superficial lesions because of the limited penetration of laser light into brain tissue (52, 53). This challenge is overcome in SDT, which employs a low-intensity ultrasound, offering superior tissue penetrance (54). SDT combines focused ultrasound with sonosensitizers, which sensitize cells to sound-induced destruction, minimizing adverse events and maximizing target responses (48, 55). Examples of sonosensitizers include 5-ALA, ATX-70, and Hypocrellin (56–58). The efficacy of SDT has been shown in studies by Sheehan et al., which demonstrated SDT’s efficacy in rat C6 and human U87 glioma cells by showing FUS and 5-ALA-induced cell death through ROS generation (59). These findings were further validated in other experiments demonstrating that focused ultrasound combined with systemic 5-ALA effectively treated gliomas in rodent models (60–63).

Overcoming the blood–brain barrier (BBB) remains a critical challenge in SDT, as most sonosensitizers cannot cross it. As a result, other studies have investigated the possibility of combining LIFUS with BBB modifiers, such as microbubbles, to increase the permeability of the BBB and improve SDT efficacy (64–66). Sonosensitizers used in Sonodynamic Therapy (SDT) comprise benign molecules that induce cytotoxic effects under an acoustic field (56). Several of these molecules are similar to those used in photodynamic therapy and are usually porphyrin-based or related compounds such as protoporphyrin IX and hematoporphyrin, among others. Emerging evidence suggests these molecules generate ROS upon exposure to ultrasound waves (67). *In vitro* investigations by Shen et al. demonstrated the efficacy of sinoporphyrin sodium, derived from photofrin II, as a sonosensitizer, showcasing significant antitumor effects on human glioblastoma cell lines (67) Particularly noteworthy was this sonosensitizer’s ability to infiltrate cancer cells and accumulate within mitochondria, thus instigating cytotoxicity via ROS production (67).

It is crucial to highlight that despite their preferential uptake by tumors, these agents exhibit considerable hydrophobicity, which results in ubiquitous distribution (68). However, as postulated by Raspagliesi et al., for cytotoxic effects to manifest in any tissue, three concurrent events must occur: (1) ultrasound administration, (2) sonosensitizer administration, and (3) the presence of a lesion where the latter attains significant concentration. Consequently, our current acceptance of SDT’s non-invasiveness towards normal brain tissue resulted from this concept, which suggests that the accumulation of sonosensitizer in healthy tissue without the other two concurrent events will render it inconsequential in the healthy tissue (69). Thus, the ideal sonosensitizer selection is crucial in SDT and should demonstrate high tumor cell affinity, prolonged neoplasm retention, and minimal impact on healthy brain parenchyma (70–72).

### 4.2 Histotripsy

Histotripsy is a non-thermal HIFUS technique that presents a promising avenue for mechanical ablation of brain tissue and tumors with precise localization, devoid of thermal effects (73). This technique employs short-duration, high-amplitude ultrasound pulses to induce acoustic cavitation within tissues, which results in inward erosion at tissue-liquid interfaces and liquefaction in dense tissue (74–76). The liquefaction process forms acellular debris, which is then gradually resorbed by the body over several months (77). Histotripsy differs from earlier thermal techniques like shockwave therapy and HIFUS because it produces more precise ablations with well-defined margins, minimizing damage to surrounding healthy tissue (14, 78).

The short duration of histotripsy ultrasound pulses restricts cavitation to the focal zone of interest, preventing extraneous tissue damage and allowing for precisely targeted ablations (79–81). This is achieved by forming dense cavitation “bubble clouds” at the focal point, which generates mechanical shearing forces and stress in the target tissue, causing cell disintegration and extracellular matrix fragmentation within those target tissues (74, 82). Cavitation migration is notably hindered outside the focal region due to insufficient amplitude to sustain dense bubble cloud formation in the off-target sites (83). The ability of histotripsy to produce clear margin lesions with minimal complications in cortical tissue has been demonstrated in detail in porcine models, suggesting its potential in brain tumor treatment (73).

Recent studies have highlighted that the acellular debris resulting from histotripsy-induced liquefaction contains tumor antigens, damage-associated molecular patterns, and heat shock proteins, potentially stimulating a tumor-specific cytotoxic T-cell response (84). Additionally, histotripsy may elicit inflammatory responses involving macrophages and B-cell lymphocytes, as evidenced in melanoma and hepatocellular carcinoma preclinical studies (74). Qu et al. showed that histotripsy in mice with melanoma or hepatocellular carcinoma not only stimulated local tumor infiltration by immune cells but also stimulated inflammation at other tumor sites not targeted by histotripsy (14, 85). While these results are promising, it is important to highlight that this study was not conducted in gliomas. Thus, whether a similar inflammatory response will be replicated in glioma remains to be determined. Further work is needed to delineate the role of histotripsy in glioma treatment.

## 5 Challenges associated with FUS

The evolving landscape of FUS holds promise for improving therapeutic outcomes through thermal ablation and novel treatment modalities, including focal BBB disruption for drug delivery enhancement. Yet, substantial challenges persist in achieving consistent and clinically meaningful outcomes. Historically, inadequate visual monitoring, thermometric control, and precise focal point determination were predominant challenges faced with FUS utility in managing CNS conditions. Advances in the utility of stereotactic skull frames, MRI-guided imaging, and thermometric monitoring have helped address these challenges (49). Though MRI-guided FUS is advantageous over Ultrasound-guided FUS because it provides a superior resolution, it should be noted that thermal ablation may interfere with MRI resolution. MRI-based acoustic radiation is a novel tool that may be useful in limiting the effects of thermal ablation (86, 87).

Further technical difficulties have been reported with FUS use, such as skull and scalp heterogeneities. These may attenuate US propagation to the target location, impeding the desired temperature for an ablative effect. Utilization of lower frequencies aids in addressing this issue; however, lowering the frequency may also induce tissue damage by cavitation. Furthermore, the translation of findings from animal studies to human clinical trials must be carefully analyzed, mainly since the human skull is thicker and harder than that of rodents. Hence, US wave attenuation is expected to be more significant in humans (87).

While FUS is a promising tool, a recent study investigating the risk of bias in animal studies and non-randomized clinical brain tumor trials showed a high risk of bias, methodological inconsistencies, and significant ethical limitations in animal and human brain tumor studies (88). Given the increasing popularity of FUS use in treating several CNS clinical conditions, it is paramount that a global initiative is established to standardize research methodologies and uphold stringent ethical norms.

## 6 Conclusion

Glioma/glioblastoma is a life-altering diagnosis for the patient. Significant advancements in developing new therapeutics to treat glioma and glioblastoma have flourished. However, these advancements have been halted by the inability of a vast number of these therapies to cross the BBB/BBTB. FUS serves as an emerging non-invasive treatment modality that could address enhanced drug delivery across BBTB and/or be used in conjunction with radiosurgery or surgical resection to improve outcomes in patients diagnosed with glioma/glioblastoma. While several ongoing clinical trials are exploring the role of FUS in brain tumors (i.e., enhanced drug delivery and tumor ablation), data on FUS use in treating spinal cord tumors is lacking. Further investigation is required to address microbubbles’ type and administration route and the FUS’s short- and long-term impact on the host immune response profile.

## Author contributions

DN: Writing – original draft, Writing – review & editing. DO-Y: Writing – original draft, Writing – review & editing. FF: Writing – original draft, Writing – review & editing. WB: Writing – original draft, Writing – review & editing. MM: Writing – original draft, Writing – review & editing.
